# “No-one just does drugs during business hours!”: evaluation of a 24/7 primary needle and syringe program in St Kilda, Australia

**DOI:** 10.1186/s12954-024-00960-9

**Published:** 2024-02-24

**Authors:** Shelley Walker, Michael Curtis, Amy Kirwan, Rebecca Thatcher, Paul Dietze

**Affiliations:** 1 National Drug Research Institute, Curtin University, Perth, Western Australia Australia; 2 Burnet Institute, Melbourne, Victoria Australia; 3 School of Public Health and Preventive Medicine, Monash University, Melbourne, Victoria Australia; 4 Access Health & 24/7 Needle and Syringe Program, Alcohol, Drugs and Other Addictions Stream, Victoria Social Mission Department, The Salvation Army Australian Territory, Melbourne, Victoria Australia; 5 Monash Addiction Research Centre, Monash University, Melbourne, Victoria Australia

**Keywords:** Primary needle and syringe programs (NSPs), Blood-borne virus (BBV) prevention, People who inject drugs, Street-based sex work, Harm reduction, Australia

## Abstract

**Background:**

Primary needle and syringe programs (NSPs) have been integral for the prevention of blood-borne virus (BBV) transmission among people who inject drugs. Despite this, many people who inject drugs face barriers accessing these services, particularly after-hours when most services are closed. To our knowledge, the St Kilda NSP, in Melbourne, Victoria, is the only primary NSP providing 24/7 dedicated stand-alone face-to-face services for people who inject drugs in Australia. We conducted an evaluation of the St Kilda NSP to assess its role and effectiveness in meeting client needs.

**Methods:**

Mixed research methods were used to conduct the evaluation. We analysed four quantitative data sets including the Victorian Needle and Syringe Program Information System data; NSP ‘snapshot’ survey data; and St Kilda NSP records of after-hours contacts and naloxone training events. Qualitative interviews were conducted with 20 purposively selected NSP clients, which were focused on individual needs, expectations and experiences accessing the service. Interviews were audio recorded and transcribed, and data were analysed thematically. A convergent research design was used to merge the five data sets.

**Results:**

St Kilda NSP had 39,898 service contacts in 2018; 72% of contacts occurred outside business hours. Similarly, of 1,185,000 sterile needles and syringes dispatched, 71% were distributed outside business hours. Participants described valuing the after-hours service because drug use patterns did not always align with standard NSP opening hours and after-hours access afforded anonymity when collecting injecting equipment. Narratives highlighted several additional benefits of the 24/7 service, including: access to safer sex equipment; material support; naloxone training; referrals to specialist services; face-to-face emotional and social support from a non-judging worker; and for women involved in sex work in particular, being able to seek refuge when feeling unsafe on the streets.

**Conclusions:**

Our study provides evidence of the social and health benefits (beyond that of preventing BBV transmission) that can be gained through the provision of 24/7 primary NSP services. Findings support the need for the establishment of after-hours primary NSPs in other areas of Australia where active street-based drug markets operate outside business hours and concentrated numbers of people who inject drugs live and spend time.

## Background

Needle and syringe programs (NSPs) have been providing harm reduction services for people who inject drugs in Australia for more than three decades [1], often cited as central in averting the HIV epidemic among this population group [2, 3].

Between 2020 and 2021 a network of more than four thousand NSPs were operating across Australia via a range of service models, distributing around 50.2 million sterile needles and syringes (herein referred to as needles/syringes) to people who inject drugs [4]. Over 100 primary NSPs provide stand-alone staffed specialist services, and approximately 800 secondary NSPs operate within existing health services (e.g. community health centres and hospitals) [4]. Some primary and secondary NSPs provide services via peer-to-peer distribution and mobile outreach services. Furthermore, needles/syringes are distributed at no cost or for a small fee via more than 2900 pharmacy NSPs and approximately 400 syringe dispensing machines (SDMs) [4].

Despite the widespread success of NSPs in averting the transmission of blood-borne viruses (BBVs) and other harms associated with injecting drugs [3, 5–7], many people who inject drugs continue to face challenges accessing needles/syringes [8–12]. Barriers accessing injecting equipment include: NSPs operating in inconvenient locations (e.g. too far to travel, poor public transport options); financial barriers accessing needles/syringes from pharmacies and SDMs; stigmatising service provider attitudes; and being unable to store sufficient quantities of syringes or carry them on their person (e.g. due to homelessness or fear of police harassment or surveillance) [8–10, 13]. An additional frequently cited barrier to accessing needles/syringes is that most government-funded primary NSPs are only open during weekday business hours, despite that injecting practices are not always planned and often occur outside these hours [2, 9, 11, 12]. For example, one Melbourne-based study found that of 584 participants who inject drugs, 23% of heroin use and 50% of methamphetamine use occurred outside of business hours, as did injecting for those who were currently employed [12]. Another study [9] found operating hours were the second most frequently reported perceived barrier for accessing NSPs (amongst a list of 16 barriers) for people who use drugs but never or infrequently used NSPs, with 57% of infrequent NSP users reporting operating hours as a barrier to access.

It is well established that SDMs address many of the barriers identified above, including enhancing geographical reach in areas of low NSP coverage, affording people privacy and anonymity when accessing needles/syringes, and increasing after-hours access to needles/syringes when fixed site secondary and primary NSPs are closed [7, 14–16]. For example, the study of SDMs in Melbourne between 2017 and 2020 cited above found 69% of 90,488 unique SDM presentations occurred out of NSP operating hours [17]. Similarly, two studies of SDM use in Sydney found over half the participants used the SDM outside of business hours with usage peaking on weekends [11, 13].

Nonetheless, studies have also found that SDMs are sometimes broken, jammed or empty [13, 18] and, when they do work, they cannot address the accompanying co-occurring health and well-being needs experienced by people who inject drugs [19–23]. Primary NSPs, on the other hand, which are funded to provide targeted face-to-face services by dedicated NSP staff, usually have the capacity to provide people who inject drugs with this additional support, including referrals to other appropriate agencies for specific presenting needs. Furthermore, primary NSPs are often a first point of contact with the health system for stigmatised populations of people who inject drugs [8, 19–22], and yet most are only open during business hours ), limiting access.

To our knowledge, the St Kilda NSP is the only primary NSP that provides 24/7 face-to-face services in Australia. In 2018, the Salvation Army commissioned the Burnet Institute to conduct an evaluation of the 24-h NSP, to determine its role and effectiveness, in the context of an application for funding to maintain the 24/7 staffed model [24]. In the following, we present findings of this evaluation, with the aim of describing what were assessed to be the multi-faceted benefits such a service can provide for people who inject drugs. To provide context, we describe the St Kilda NSP and its geographical location, followed by the methods used to collect and analyse data, the research sample, and results. We conclude with a discussion of the implications of our analysis.

## Research setting and context

St. Kilda, where the 24/7 NSP is located, is a suburb approximately 6 km south of the Central Business District of Melbourne. Between the 1950s and 1990s the area was characterised by deteriorated building stock, active street drug trades, sex work and homelessness [25, 26]. The process of gentrification and urban redevelopment began around three decades ago, leading to rising property values and an associated decline in low-cost accommodation [26]. Anecdotally, these changes have led to increased pressure on low-income and disadvantaged residents—including people who use drugs, people experiencing homelessness and people involved in sex work—which has resulted in increased demand for health and community services to meet their needs. To our knowledge, no published data exist about gentrification-induced displacement in St Kilda; however, studies in other geographical locations have found links between gentrification, unstable housing, and homelessness [27, 28]. Furthermore, anecdotal evidence suggests ongoing gentrification in St Kilda has driven a smaller low-cost rental market and contributed to the closure of high-volume low-cost rooming houses (such as the Gatwick Hotel [29]), potentially increasing housing instability and homeless in the area. Despite this gentrification, a significant street-based drug market and street-based sex industry continue to exist in St Kilda.

The St Kilda primary NSP was one of the first government-funded fixed site primary NSPs in Australia. Established in March 1991 amidst a growing concern about the spread of BBVs, particularly HIV/AIDS, the NSP was immediately well utilised, providing over 8000 needles/syringes in the first month of its operation [30]. The service was co-located with The Salvation Army’s Crisis Contact Centre in St Kilda and, at the time, relied solely on word of mouth to promote its existence. Following a successful proposal to the Victorian Government to expand the operating hours of the NSP, a 24/7 service delivery model was introduced in 2008. In 2024, the NSP continues to be staffed all day every day of the week, including on public holidays.

The NSP remains co-located within The Salvation Army’s Crisis Contact Centre, which operates a state-wide after-hours emergency accommodation response service (including housing advocacy, information, support, material aid and referral services for people experiencing or at risk of homelessness). The NSP is operationally managed by a specialist AOD harm reduction Primary Health Care service (Access Health), located in the building next door. Access Health is funded by the Victorian Government to provide a local Comprehensive Primary Health Care response to people who inject drugs and/or people who experience barriers accessing mainstream and specialist health services due to experiences of homelessness, interactions with the justice system or engagement in street-based sex work. The integrated service model consists of six organisations co-located under formal partnership agreements to provide a ‘one-stop-shop’ of accessible, flexible and non-judgemental health and specialist services. A range of other services are provided by Access Health, including BBV testing and treatment; harm reduction services (e.g. opioid agonist treatment, overdose education, free naloxone distribution and training); nursing services (e.g. pathology, wound care, outreach and general health); drug and alcohol intake, assessment and counselling services; forensic psychology; dental services; sexual health testing and treatment; chronic disease management; and services for Aboriginal and Torres Strait Islander Peoples. Primary functions of the NSP include providing sterile injecting equipment (including filters, swabs, disposal containers, tourniquets and mixing spoons); services for the disposal of used equipment; harm reduction information and education; safer sex equipment (SSE, e.g. condoms, dams, personal lubricant); and referral to specialist health and support services.

The NSP is overseen and led by an experienced senior social worker, and most NSP workers have a tertiary qualification or equivalent in a specific or related field, including social work, and alcohol and other drug counselling. Lived and living experience is held as a highly valued staff skill set. NSP workers receive on-the job training, including education about: drugs, drug use and harm reduction practices; the needs of the different cohorts accessing the service; and processes and services for referring clients. No external signage exists at the NSP, to enhance anonymity. A private space behind a closed door allows one client at a time to enter and engage with a NSP worker, which is designed for private individual one-on-one conversations, unless clients request and consent to enter in small groups (e.g. with friends, partners). Injecting equipment and most other resources (excluding SSE and safe disposal containers) are kept behind the counter, so clients must request what they need.

## Methods

A mixed methods research design was used to conduct our study, with the aim of capitalising on the complementary strengths of both quantitative and qualitative methods [31], to provide a more comprehensive understanding of the role and effectiveness of the St Kilda NSP for individuals using the service.

The research was informed by an advisory group which included staff from the St Kilda NSP, Salvation Army senior staff, representatives from the Victorian Government and members of the research team. Ethics approval to conduct the study was granted by the Alfred Health Human Research Ethics Committee (Approval #501-18).

### Quantitative study

Four quantitative data sets were used for our analysis. For the period 1 January 2018 to 31 December 2018, we sourced NSP Information System (NSPIS) data (*n* = 39,898), which routinely captures self-report demographic, harm reduction service activity and survey data of needle distribution and returns from NSPs across the state of Victoria [32]. At the time of data collection, St Kilda 24-h NSP counted needles distributed as the number of tips/needles distributed per service contact. Accordingly, a service contact which included distribution of 10 × 27-gauge syringes (needle tips and barrels) along with 10 × 19-gauge needle tips was recorded as 20 needles distributed. The same counting rules applied to needles returned to the NSP. NSPIS does not include data on the types of needles distributed/returned at each service contact.

We also used complementary NSP ‘snapshot’ survey data, which was sourced via a series of brief additional questions added to the St Kilda NSPIS administrative questionnaire for the purposes of the St Kilda evaluation; responses were captured during each service contact (*n* = 3218). Snapshot surveys were conducted in three brief periods between October and November 2018 (Snapshot Survey 1: 13:00 on 12/10/18 to 11:00 on 24/10/18; Snapshot Survey 2: 12:00 24/10/18 to 16:00 on 07/11/18; Snapshot 3: 16:00 07/11/18 to 12:00 on 14/11/18). Participants were questioned about the drug they last injected, with the remaining questions varying across surveys to capture information of interest to the evaluation, as detailed in Table 1. Additionally, the Salvation Army provided two records of additional activities of the NSP, including a record of additional after-hours services (5 pm–8:50 am) for the period between 1st July 2016 and 30th November 2018 and a record of naloxone training events (which included the distribution of vouchers to obtain naloxone, to training participants) for the period 1st February 2018 to 30th November 2018.

**Table 1:** Results of NSP snapshot surveys

	Business hours^a^	Evening^b^	Late night^c^	W/ends and public holidays^d^	*p* value
	*n* (%)	*n* (%)	*n* (%)	*n* (%)	
*Snapshot surveys 1–3 (N = 3218)*					
Drug last injected					
Heroin	390 (32)	279 (23)	184 (15)	356 (29)	< 0.001^f^
Methamphetamine	428 (31)	316 (23)	302 (22)	357 (25)	
PIEDS^e^	59 (38)	53 (34)	15 (10)	27 (18)	
Pharmaceuticals^g^	62 (42)	13 (9)	36 (25)	35 (24)	
Other^h^	24 (46)	12 (23)	0 (0)	16 (31)	
Cocaine	9 (4)	21 (10)	np^i^	10 (5)	
Missing	61 (29)	64 (30)	24 (11)	64 (30)	
*Snapshot survey 1*^**j**^* (N = 1000)*					
Aboriginal and Torres Strait Islander					
No	297 (35)	198 (24)	138 (16)	205 (24)	0.603^f^
Yes	42 (37)	28 (25)	11 (10)	33 (29)	
Missing	18 (38)	13 (27)	6 (13)	11 (23)	
LGBTQIA + ^k^					
No	278 (35)	190 (24)	130 (16)	200 (25)	0.151^f^
Yes	52 (38)	38 (28)	18 (13)	30 (22)	
Unsure	11 (69)	0 (0)	np^i^	np^i^	
Missing	16 (33)	11 (23)	6 (13)	15 (31)	
Parents born in Australia					
Both born in Australia	200 (36)	131 (23)	93 (17)	139 (25)	0.284^f^
One born outside Australia	48 (30)	42 (26)	32 (20)	38 (24)	
Both born outside Australia	78 (38)	48 (24)	24 (12)	54 (26)	
Unknown	10 (50)	7 (35)	0 (0)	np^i^	
Missing	21 (40)	11 (21)	6 (11)	15 (28)	
*Snapshot survey 2*^*l*^* (N = 1533)*					
Employment status					
Unemployed	214 (30)	155 (22)	162 (23)	177 (25)	< 0.001^f^
Employed	116 (27)	143 (34)	74 (17)	92 (22)	
Pensioner	88 (39)	43 (19)	28 (12)	68 (30)	
Student	7 (28)	6 (24)	4 (16)	8 (32)	
Other	10 (31)	6 (19)	8 (25)	8 (25)	
Missing	20 (17)	49 (42)	8 (7)	39 (34)	
Accommodation type					
Private rental	106 (26)	112 (28)	79 (19)	109 (27)	< 0.001^f^
Public housing	117 (36)	64 (20)	79 (25)	62 (19)	
NFA^m^/homeless	79 (28)	71 (25)	55 (20)	75 (27)	
Owner occupied	47 (32)	53 (36)	18 (12)	28 (19)	
Boarding/rooming house	55 (38)	17 (12)	24 (16)	50 (34)	
Living with parents/family	18 (24)	25 (33)	13 (17)	19 (25)	
Crisis/supported/transitional/temporary	10 (26)	12 (31)	8 (21)	9 (23)	
Other	np^i^	np^i^	np^i^	np^i^	
Missing	20 (18)	47 (43)	5 (5)	37 (34)	
Accommodation stability					
No	144 (31)	103 (22)	95 (20)	122 (26)	< 0.001^f^
Yes	291 (30)	251 (26)	183 (19)	235 (24)	
Missing	20 (18)	48 (44)	6 (6)	35 (32)	
Felt unsafe in the last 24 h					
No	388 (32)	294 (24)	239 (20)	286 (24)	< 0.001^f^
Yes	45 (21)	61 (28)	38 (18)	72 (33)	
Missing	22 (20)	47 (43)	7 (6)	34 (31)	
*Snapshot 3*^*n*^* (N = 685)*					
Length of NSP use (months; [median; IQR^o^])	84 (36–180)	78 (36–180)	60 (12–96)	120 (48–180)	< 0.001^p^
Freq. of NSP use (per/wk; [median; IQR^l^])	1 (0–5)	1 (0–3)	2 (1–3)	2 (1–4)	< 0.001^m^
Referral to health/support service					
No	72 (22)	79 (24)	70 (21)	109 (33)	< 0.001^f^
Yes	124 (42)	25 (9)	51 (17)	93 (32)	
Missing	25 (40)	13 (21)	2 (3)	22 (35)	
Attended referral (*N* = 293)					
No	21 (51)	4 (10)	11 (27)	5 (12)	0.012^f^
Yes	102 (41)	19 (8)	40 (16)	86 (35)	
Missing	np^i^	np^i^	np^i^	np^i^	

^a^Business hours: 09:00–16:59; ^b^evening: 17:00–22:59; ^c^overnight: 23:00–08:59; ^d^weekend: 00:00 Saturday–23:59 Sunday and public holidays 00:00–23:59; ^e^PIEDs: performance or image-enhancing substances, e.g. steroids, melanotan; ^f^Chi-square test; ^g^extra medical use of prescribed medications, e.g. oxycontin, buprenorphine, dexamphetamine; ^h^includes ketamine, MDMA, GHB and cocktailed heroin and methamphetamine; ^i^np: value not provided as cell size < 5; ^j^Snapshot Survey 1 time period: 13:00 on 12/10/18 to 11:00 on 24/10/18; ^k^lesbian, gay, bisexual, transgender, queer, intersex, asexual and more; ^l^Snapshot Survey 2 time period: 12:00 24/10/18 to 16:00 on 07/11/18; ^m^no fixed address; ^n^Snapshot Survey 3 time period: 16:00 07/11/18 to 12:00 on 14/11/18; ^o^IQR: interquartile range; ^p^Kruskal–Wallis test

Descriptive statistics were generated for quantitative variables relating to socio-demographics and experiences of substance use, health outcomes and service use. The numbers of needles/syringes and SSE distributed, and used needles returned, and client demographics were analysed by client gender and/or time of service use. Data analysis was conducted using Stata 16.1.

### Qualitative study

Qualitative interviews were conducted with 20 clients of the NSP between October and November 2018. Participation was voluntary and involved informed consent. Interviews were guided by a semi-structured interview schedule focused on participants needs, expectations and experiences in relation to accessing the service, and any impact the service had had on their lives. Purposive sampling was used to ensure the inclusion of information-rich cases, including an intention to recruit at least some participants who had been accessing the service for at least five years.

Twelve women and eight men who used the NSP service participated in qualitative interviews; participants were aged between 28 and 61 years. Most described experiences of periods of homelessness, unemployment, poor mental health and ongoing challenges with drug use. A long history of attending the NSP was common for most participants, with some having attended the service since it first opened. Many participants resided in the local area or had done so in the past; others reported working in the area.

Interviews were audio recorded and transcribed, and data were managed using NVivo qualitative data software [33]. Data were analysed thematically using a coding framework. Extracts of transcripts were assigned to deductive codes based on topics within the interview schedule, and as new patterns, commonalities and discrepancies were identified, additional codes and sub-codes were inductively created to represent these themes [34]. All authors agreed upon four final themes. To increase the validity of our findings, we used a convergent mixed methods design to integrate the data [35]. The approach involved weaving the quantitative and qualitative data together into three of the four themes we identified (excluding “NSP client demographics and service use”, which only includes quantitative data).

## Results

Results are presented together via four themes: 1) St Kilda NSP client demographics and service use; 2) Accessing needles/syringes after-hours; 3) Addressing women’s after-hours needs; and 4) More than a service for accessing needles/syringes.

To protect the identity of participants, quotes are followed by a unique identifier and the sex of the participant (i.e. P#, male/female).

### St Kilda NSP client demographics and service use

In 2018, according to NSPIS data, 39,898 service contacts occurred for the NSP; most contacts were for males (73%), typically over 36 years of age (65%). Table 1 details characteristics of snapshot survey respondents, with data on last drug injected aggregated across all three snapshot surveys with the remaining variables unique to each survey. Snapshot survey data revealed approximately 11% of service contacts were for people who identified as Indigenous and 16% who identified as lesbian, gay, bisexual, transgender, queer or intersex (LGBTQI). Almost half (43%) stated that one or both parents were born outside Australia, almost half (46%) were unemployed, and around one in five were living in public housing (21%) or were homeless (18%).

According to snapshot survey data, methamphetamine was the most frequently injected substance (44%), followed by heroin (38%). People attending the service overnight were more likely to report recent methamphetamine injection. A small number of clients reported performance and image-enhancing drugs (e.g. steroids or melatonin) as their drug most recently injected.

Most NSP visits occurred outside of business hours (i.e. 09:00–16:59), with 19%, 19% and 32% of visits occurring during evening hours (17:00–22:59) late night hours (23:00–08:59), and weekends and public holidays, respectively. Times of presentation were similar across NSPIS data and snapshot survey data. Analyses of 2018 NSPIS data (see Table 2) found different patterns of service access according to sex, with 75% of visits by females occurring outside of business hours, compared to 69% for men (*X*^2^ [*df* = 1, *n* = 39,726] = 147.9527, *p* ≤ 0.001).

**Table 2:** Sex, number of sterile needles distributed, number of needles returned and visits including distribution of SSE by time of NSP visit between 1st January 2018 and 31 December 2018 (*source*: NSPIS)

	Business hours^a^	Evening^b^	Late night^c^	Weekends and public holidays^d^	*p* value
Sex					
Male	9091 (31)	5487 (19)	5154 (18)	9247 (32)	< 0.001^e^
Female	2697 (25)	2223 (21)	2244 (21)	3583 (33)	
Missing	65 (38)	34 (20)	14 (8)	59 (34)	
Sterile needles distributed	345,119 (29)	252,874 (21)	212,142 (18)	374,865 (32)	< 0.001^f^
Needles returned	148,061 (31)	102,282 (21)	90,166 (19)	144,007 (30)	0.001^f^
SSE distributed	651 (14)	929 (20)	1449 (31)	1717 (36)	< 0.001^e^

^a^business hours: 09:00–16:59; ^b^evening: 17:00–22:59; ^c^overnight: 23:00–08:59; ^d^weekend: 00:00 Saturday–23:59 Sunday and public holidays 00:00–23:59; ^e^Chi-square test; ^f^Kruskal–Wallis test

### Accessing needles/syringes after-hours

According to 2018 NSPIS data, most clients accessed the NSP to obtain injecting equipment. The NSP distributed a total of 1,185,000 needles during 2018; almost three quarters (71%) were distributed outside of business hours (see Table 2). Similarly, of the needles/syringes that were returned to the service (*n* = 484,516), 69% were returned outside of business hours. Seventy-five per cent of sterile needles were distributed to males who made up 71% of total service visits. Needles/syringes were mostly obtained from the NSP in amounts of five or less (44%), followed by 6–10 (25%) and 100+ (15%).

Many interview participants described valuing the after-hours nature of the NSP service for collecting injecting equipment because their drug use patterns did not always align with standard NSP opening hours (9–5 pm weekdays).

Well, no one does drugs just during business hours. It’s an impulse thing. (P3, male)

…like anyone who is a drug user, like we can prioritise […] but things can just go out of whack just like that, and all of a sudden at the drop of a hat, you’ll go “bang” and you’re off on a mission. Yeah, so it’s really hard to be scheduled and go, “okay it’s only open from 9 am until whatever”- it just doesn’t work like that. (P7, female)

For people who worked regular business hours (9 am–5 pm), being able to access needles/syringes after-hours was also considered an important factor.

It’s silly to think that people will just use or inject 9–5 pm. You know there’s a lot of people who like myself work full-time. I think people don’t realise that. I actually think it’s at night-time that it’s needed most. (P18, male).

Although some participants acknowledged that SDMs provided access to needles/syringes after-hours, they were described as empty on some occasions or required a fee to purchase items, which created access barriers.

[There’s] a vending machine closer for me [but] most of the time the free lot are gone, and the next lot cost a gold coin. But if the slot is full, you can’t get any. So, you risk not being able to get needles at all. (P6, female)

Mobile NSPs were raised by interview participants as an important service operating out of business hours, but as one participant highlighted, these services (in Melbourne) do not deliver injecting equipment past late evening hours.

There’s foot patrol (mobile NSP) but that has really strict hours as well – they don’t have the funding unfortunately, and sometimes you need to use outside of those hours. (P5, male)

One interviewee acknowledged his appreciation of the extended opening hours of the Melbourne Supervised Injecting Room, which he often visited to inject safely. He reflected, however, on how it was not open overnight and on weekends when he sometimes most needed to access needles/syringes.

Well, [the St Kilda NSP] is the only one apart from the safe injecting rooms - which I use … But as the night rolls in - I think it’s [open until] 7 pm on weekdays and 5 pm on the weekends – so a lot of times it’s not open when I need it. (P1, male)

Most participants viewed the 24-h operation of the NSP as vital to their ability to protect themselves from BBVs and other injecting-related injury and disease. In doing so, they expressed a sophisticated understanding of the ‘return on investment’ the 24-h NSP service can provide, by not being forced to reuse and/or share injecting equipment if the service was closed.

It’s because of night-time needle exchanges that I’m lucky enough not to be HIV positive. (P7, female)

Well, you don’t have to worry about using dodgy fits, so your health is protected. I don’t know where I’d go to get a ‘freshy’ [sterile needle/syringe] if this place wasn’t open. (P2, female)

One participant who had experienced periods of homelessness and sometimes injected drugs at night, described how when he was homeless the NSP was a saviour because he had no place to store boxes of needles/syringes.

I come all hours of the day and night - no specific times, just when I ‘get on’ [inject drugs]. When I've got a home, I tend to keep a box of ‘fit’s [needles/syringes] at home but when I’ve been homeless it’s been so helpful to [get them] after midnight or even after 5 pm. (P6, male)

Being able to collect injecting equipment anonymously, when “it was dark” and deemed less likely to be seen by on-lookers, was also acknowledged as a benefit of the NSP, especially for avoiding stigma and discrimination.

At night-time if you do like use and you want to be discreet about it, like you can be, but during the day people obviously know that you’re coming in and out – and then you constantly get labelled you know – “junkie”. (P9, female)

### Addressing women’s after-hours needs

Women represented almost a third of all clients visiting the St Kilda NSP after business hours, with an increase overnight of 7% compared to access during business hours. Some of these women (including transgender women) worked in the sex industry and accessed the service for injecting equipment and/or SSE; however, many also used the service to seek refuge when feeling unsafe on the streets, to seek warmth in the winter months, or to access support from a non-judging worker.

**Fig. 1:**
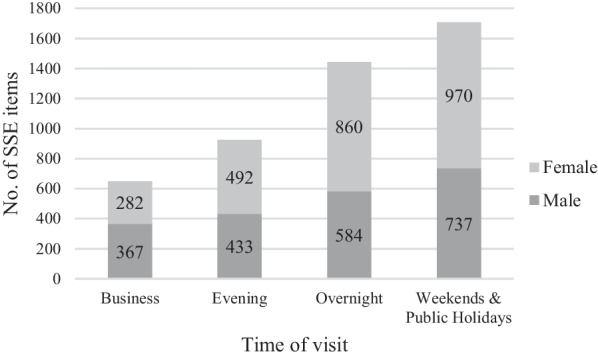
Visits including distribution of SSE* by time of visit between 1st January 2018 and 31 December 2018 (*source*: NSPIS)

Overall, females were more likely to visit the NSP for SSE collection than males in 2018; however, this varied significantly by the time of visit. Women accounted for 43% (*n* = 282) of SSE distributions during business hours, but most SSE distributions at all other times (see Fig. 1).

People engaged in sex work described the importance of having free condoms available after-hours to maximise opportunities for safer sex while working.

If we couldn’t access free condoms, then there’d be girls having to do things they don’t want to do […] because a lot of the men won’t go and buy [condoms] because they can get sex without a condom, so you’re pretty much fucked. (P20, female)

With the NSP I mostly drop in around 10–11 pm at night or 3–4 am in the morning to get condoms - that’s when I tend to come out for night times, and usually at the end of a shift […] so middle of the night times. (P9, female)

Some women believed if the St Kilda NSP was not open overnight they would be forced to obtain SSE through illegal means.

Because we can get what we need we don’t need to steal like condoms and stuff. (P16, female)

When I first came to St Kilda and worked, if these guys weren’t here, I would’ve been in jail because I would’ve been stealing condoms and stuff, and it would have just tipped my criminal record over the edge. (P13, female)

Although the numbers of people engaged in sex work were not as high as some other groups accessing the NSP, the safety role it provided for this cohort (e.g. from male perpetrators of violence or males who posed a potential threat to their safety on the streets), was considered one of the critical aspects the service. For these reasons, the NSP was often described as a temporary refuge.

If you’re wary on someone, like if you’re out there and you’re on your own, and you can see a car you know … Like sometimes I get wary on someone, so I’ll just come in and I go, “I’m just gonna sit in here for five minutes”. (P20, female)

A lot of the working girls will run in there for sanctuary, sometimes because some predator will be stalking them. I know for me, on many occasions - cos I’m only little - quite often a couple of guys will see me and go, “Yeah, she’ll have stuff [illicit drugs] on her for sure, let’s get her, she’s on her own”. That’s a regular occurrence in St Kilda, a lot of full-on stand-overs! Yeah, this place is really a little sanctuary, but especially because it’s open 24/7. (P19, female)

The local street knowledge of NSP workers and access to the monthly “Ugly Mugs Report”^1^, was considered highly valuable and appreciated by participants, especially for those who were engaged in sex work.

The workers know so much about what’s happening on the streets […] Yeah, the girls will go in and talk about what’s going on, so the workers will hear about ‘mugs’ [men often known to the police as posing a safety threat], or they’ll hear about gear, or they’ll hear about issues with cops and then they’ll pass that information on to us. So, it’s a really good touchstone for me. (P14, female)

A couple of participants described how they felt the attitude of some local police towards women engaged in sex work had improved because of the work of the NSP to educate police.

… the cops and the exchange and the girls, like everybody liaises - and the cops, because of this, I think [it has] kind-of changed their attitude towards us. Like our safety seems to be considered more important. (P11, female)

### More than a service for accessing needle/syringes

Data collected by St Kilda NSP revealed a diverse range of services being delivered to clients after business hours (see Table 3). A total of 293 after-hours service contacts were responded to between July 2016 and December 2018, with 658 responses provided. The most common types of response were supportive chats or debriefs (*n* = 180), referrals to support services (*n* = 99), material support (*n* = 149) and basic medical assistance (*n* = 30). Just under 25% of responses were provided to clients experiencing violence, safety issues, mental health issues or who were victims of crime of various types. Most of these additional after-hours’ responses were provided to women (67%).

**Table 3:** Additional after-hours (evening, overnight and weekends) response frequencies 1st July 2016–30th November 2018 (*source*: after-hours log)

Response type	*n* (%)
Debrief, chat	180 (27)
Referral	99 (15)
Response to physical assault (inc. weapons); verbal abuse, harassment, or threats; immediate safety; stalking; intimate partner violence	97 (15)
Tea, coffee and/or food	76 (12)
Blanket and/or clothing	73 (11)
Medical and/or band-aids	30 (5)
Sanitary/hygiene products	30 (5)
Mental health support	28 (4)
Contacting police	14 (2)
Robbed1	11 (2)
Information sharing	6 (1)
Contacted sex industry service	6 (1)
Response to coercion (e.g. forced into sex or drug taking)	< 5 (–)
Ugly mug report	< 5 (–)
Total	658 (100)

Although on-the-spot referrals to most services cannot be facilitated by the NSP overnight (due to the standard 9 am–5 pm operating hours of most services), for some clients, simply having the opportunity to obtain information about services was described as useful in leading to opportunities for referral in the future.

The hours really suit me. Just knowing that it is here and knowing that any question in this field that I don’t have an answer to, about where to go or what to do, I can get it even if it’s three in the morning. (P6, male)

Many participants described the importance of material support provided without judgement, such as blankets if they were homeless or engaged in street-based sex work, food if they faced financial challenges, or referrals to housing services.

They are more than just handing out needles […] I’ve been able to get things I’ve needed for drug use and condoms [and] a lot of the time they’ve had donations like bread or vegies - just little things like that. It doesn’t seem a lot, but when you have nothing, it means a lot. [...] They knew I’d spent my money on drugs and that’s why I needed vouchers for food, but they didn’t judge. (P7, female)

… that was why it was really useful - because disasters always happen at 3 in the morning. I wasn’t even thinking at the time that they were going to help me. They just asked me what was wrong, and instantly […] they are arranging somewhere for me to go [for the] night, something for me to eat. They were straight onto it. (P8, male)

In 2018, a total of 673 people were trained to respond to opioid overdoses, including how to administer naloxone; the majority (60%) of training participants were male. Half (50%) of this training was provided outside of business hours, a factor that was appreciated by clients who accessed the training overnight.

I did the naloxone training there one night, which has saved lives. You can’t get that 24 hour a day [anywhere else] (P4, male).

Several clients discussed experiencing significant social isolation. Contact with St Kilda NSP staff represented one of their few opportunities for human interaction, particularly after-hours. That these experiences were overwhelmingly positive and valued highly was evident in participants’ accounts.

You have this night-time population of people and I imagine it happens to women [not engaged in sex work] as well. Somewhere between 40–50 [years of age] they start becoming night people and so there is a lot of people just floating around in the night that don’t get enough contact with people. (P17, female)

… how are you supposed to talk to a vending machine, asking if you’re okay. It’s the human contact that you need. I’ve come in here a couple of times when nothing else has been open like when my dog died, and they were able to talk to me about it and they care. It would be devastating if this place wasn’t open (P20, female).

Some participants felt they owed their life to having been able to attend the St Kilda NSP after-hours when needed:

… there have been times when I’ve gone out and ‘scored’ [purchased drugs] with the intention of killing myself because my depression got so bad. I’d picked up syringes and I didn’t even tell them that that was my intention cos I didn’t want the burden of them knowing that. […] That small interaction of them understanding me and offering support … I went home and I didn’t overdose on purpose. I just used a small amount instead. That’s happened more than once. In a sense you could say I almost owe my life to these people without them even knowing it. (P3, male)

I wouldn’t be here telling you this story today if it wasn’t for [them]. It’s kept me alive. It’s kept me together. It’s just kept me putting one foot in front of the other. They really give a damn. They’re so selfless. (P14, female)

Highly valued by several participants was the confidential nature of visits to the NSP. The assurance they would be seen individually, could take as much time as they needed without interruption and could visit at times that were more discreet (e.g. after dark) was described as particularly important.

… because of the one person at a time, people don’t feel threatened [and] people don’t know what you’re getting, because quite often there’s so many people, or predators out there, and they’ll be like, “Ok, given what they’re picking up [needles], they’ve obviously got a lot of gear, you know, let’s follow these people”. (P6, male)

All participants who were interviewed, without exception, talked positively about the St Kilda NSP staff.

They’re incredible people [the NSP workers]. There’s no judgement, there’s no nothing. You walk through that door at four in the morning - it doesn’t matter what time it is - there’s a friendly face giving you exactly what you need, so there’s no need to share anymore, shit like that. It’s just a very important hub that place, and I couldn’t even imagine St Kilda functioning without it. (P7, female)

## Discussion

Our evaluation of the St Kilda primary NSP suggests that there are significant social and health benefits that can be afforded through the provision of 24/7 services for people who inject drugs. Our analysis of client demographics highlights the substantial levels of disadvantage experienced by clients of the St Kilda NSP, and the services capacity to address equity of access for marginalised sub-populations of people who inject drugs, including Aboriginal and Torres Strait Islander Peoples, people who are experiencing homelessness and unemployment, people who identify as LGBTQI and people engaged in sex work. Moreover, given 71% of needles/syringes provided in 2018 were distributed outside of regular business hours, our findings demonstrate the critical need for access to injecting equipment when most other NSPs are closed [2, 4, 8, 9, 11, 15, 17, 36]. Client narratives support this, emphasising that injecting drug use does not always occur in standard opening hours, that injecting episodes are not always planned, and that for individuals who are experiencing homelessness and unable to store injecting equipment, that the 24/7 availability of individual needles/syringes is essential for their health and safety [37].

Many evaluations of NSP services have focused on understanding their effectiveness in preventing BBV transmission and related harms, for HIV and hepatitis C in particular [1, 5, 6, 21, 23, 38–43]. Our study has demonstrated that a primary 24/7 NSP can increase after-hours access to injecting equipment (for preventing BBV transmission) but can also increase after-hours access to a diverse range of additional health and social services. That is, although the service is limited in its capacity to provide immediate referrals overnight, our data highlight that after-hours access to crisis accommodation, material support and first aid are commonly provided and valued, as was the opportunity to participate in naloxone training overnight. Although most NSP service contacts were for the collection of injecting equipment and SSE, our findings suggest it also offers a place where clients can find refuge and support when no other services are available. Narratives highlight how the opportunity for human contact overnight with someone who cared and valued clients was highly regarded, especially for those who were experiencing homelessness or engaged in sex work, which was identified in large part due to the non-judgemental and welcoming attitudes of staff. Furthermore, qualitative findings highlight how the 24/7 operating hours of the NSP were able to accommodate client needs for anonymity by being able to access the service after-hours or when fewer people were around—a factor that helped avoid stigma.

Study findings also underscore the value of the 24/7 operating hours for women and people engaged in sex work. The fact that women constituted just over a quarter of service contacts yet made up almost a third of all night-time clients, underlines the importance of around-the-clock access, especially for those involved in street-based sex work. Although women make up a smaller percentage of people who inject drugs in Australia [44], they are among the most hidden [45]. They experience significant differences in their health status and injecting-related risk practices compared to men, including an increased likelihood of injection-related injuries and harms, mental health issues and physical and sexual violence [46, 47]. Those who are involved in sex work are at greater risk of sexually transmitted infections and threats of violence that undermine their ability to practice safer sex and drug use, both in contexts with intimate partners and during sex work [45, 47–49]. Women who inject drugs are also more likely to experience a greater burden of substance use-related stigma than men, due to gendered social norms [8, 50], which can be a barrier for accessing harm reduction services such as NSPs [45, 49, 51, 52]. As our findings have demonstrated, the St Kilda NSP’s capacity to respond appropriately and non-judgementally to many of the issues described above was considered one of the most important aspects of the services provided for women outside of business hours (e.g. responses to incidents of intimate partner violence, and sexual assault, harassment and violence on the streets; access to injecting equipment and SSE; and the opportunity for safety in the confines of the NSP). Women’s voices and experiences have often been silenced in studies around harm reduction service provision, hampering the development of harm reduction services to meet their specific needs [47, 53]—our study has helped fill this gap by giving voice to disadvantaged women accessing the NSP.

While access and equity are key challenges for the distribution of sterile needles/syringes to people who inject drugs, as demonstrated in this study, more nuanced models of syringe distribution can effectively respond to different time periods of needle/syringe demand and the specific health and well-being needs of disadvantaged sub-populations of people who inject drugs.

Our complimentary use of five data sets allowed us to develop a nuanced and comprehensive understanding of the role and effectiveness of the St Kilda NSP for individuals using the service. By capitalising on the strengths of both quantitative and qualitative methods we believe we have also increased the rigour and credibility of our findings [31].

### Limitations

Our study has some limitations. NSPSIS and NSP snapshot data represent service contacts rather than individual participants, and so we cannot accurately profile individual clients. Furthermore, snapshot surveys are subject to recall and social desirability biases, and we recognise that clients who participated in qualitative interviews were selected by staff who work in the NSP and thus may have been positively biased in their view of the service. Finally, we acknowledge that conducting an overall economic cost–benefit analysis of the St Kilda 24/7 NSP would have been beneficial and thus we recommend this occurs in the future.

## Conclusion

Our study provides important implications for policy makers and implementers of NSP services. By examining the role and effectiveness of the St Kilda 24/7 primary NSP service model, we have demonstrated that through the provision of after-hours services, so much more can be achieved (beyond the role of preventing the transmission of BBVs), for improving the overall health and well-being of disadvantaged sub-populations of people who inject drugs. Findings support the need for the establishment of 24/7 primary NSPs in other areas of Australia where active street-based drug markets operate and concentrated numbers of people who inject drugs live and spend time.

## Data Availability

Study data are not publicly available due to their sensitive nature and the potential for participant identification. Data may be made available for collaborative work subject to permission from the project’s Chief Investigators, including The Salvation Army Territory Australia. Researchers interested in data access should contact the corresponding author (S.W.) to discuss potential collaborations.

## Footnotes

1 The Ugly Mugs Report is published by St Kilda based “Resourcing Health and Education in the Sex Industry (RhED)” in collaboration with local police, with the aim of alerting sex workers to dangerous individuals in the local area.
